# Direct Oral Anticoagulants for Stroke and Systemic Embolism Prevention in Patients with Left Ventricular Thrombus

**DOI:** 10.3390/jpm13010158

**Published:** 2023-01-14

**Authors:** Minerva Codruta Badescu, Victorita Sorodoc, Catalina Lionte, Anca Ouatu, Raluca Ecaterina Haliga, Alexandru Dan Costache, Oana Nicoleta Buliga-Finis, Ioan Simon, Laurentiu Sorodoc, Irina-Iuliana Costache, Ciprian Rezus

**Affiliations:** 1 Department of Internal Medicine, “Grigore T. Popa” University of Medicine and Pharmacy, 700115 Iasi, Romania; 2 III Internal Medicine Clinic, “St. Spiridon” County Emergency Clinical Hospital, 700111 Iasi, Romania; 3 II Internal Medicine Clinic, “St. Spiridon” County Emergency Clinical Hospital, 700111 Iasi, Romania; 4 Cardiovascular Rehabilitation Clinic, Clinical Rehabilitation Hospital, 700661 Iasi, Romania; 5 Department of Surgery, Faculty of Medicine, “Iuliu Hatieganu” University of Medicine and Pharmacy, 400012 Cluj-Napoca, Romania; 6 Cardiology Clinic, “St. Spiridon” County Emergency Clinical Hospital, 700111 Iasi, Romania

**Keywords:** direct oral anticoagulants, left ventricular thrombus, myocarditis, hypertrophic cardiomyopathy, COVID-19

## Abstract

In recent years, direct oral anticoagulants (DOAC) have accumulated evidence of efficacy and safety in various clinical scenarios and are approved for a wide spectrum of indications. Still, they are currently used off-label for left ventricular thrombus owing to a paucity of evidence. For the same reason, there is a lack of guideline indication as well. Our work is based on an exhaustive analysis of the available literature and provides a structured and detailed update on the use of DOACs in patients with left ventricle thrombus. The safety and efficacy of DOACs were analyzed in particular clinical scenarios. As far as we know, this is the first paper that analyzes DOACs in this approach.

## 1. Introduction

In recent years, direct oral anticoagulants (DOAC) have accumulated evidence of efficacy and safety in various clinical scenarios and currently have a wide spectrum of indications. Studies of DOACs in patients with atrial fibrillation (AF) have shown that they reduce the risk of stroke and systemic embolism equal to or better than vitamin K antagonists (VKA), with a similar or lower bleeding risk, and are currently preferred over VKA in patients eligible for a DOAC. Left atrial (LA) or left atrial appendage (LAA) thrombi are identified in up to 10% of patients with AF and current guidelines recommend anticoagulant therapy for at least three weeks upon their detection [1]. Resolution of LA/LAA thrombus in patients receiving a DOAC has already been reported, even in cases with large thrombi [2,3,4]. Moreover, the X-TRA study—a comparative study between rivaroxaban and VKA—showed that rivaroxaban can be a potential option for the treatment of thrombi identified in LA/LAA in patients with AF [5]. Encouraged by this evidence, the idea of using DOAC in patients with left ventricle (LV) thrombus took shape.

**Figure 1:**
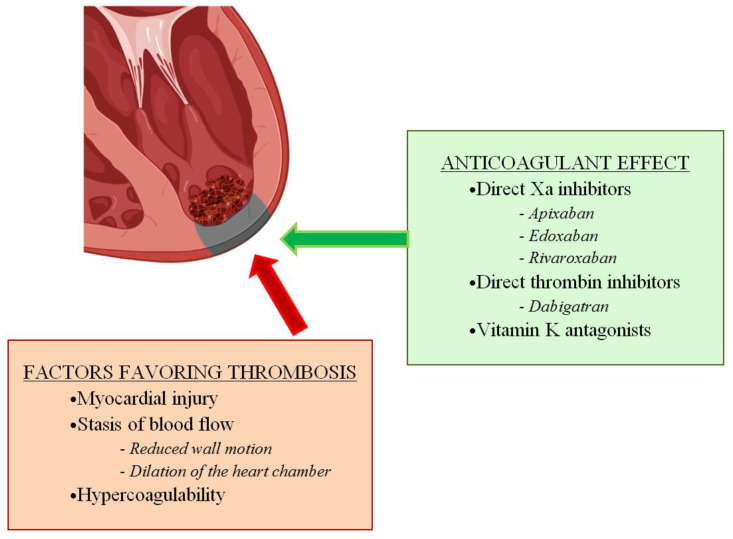
The main determinants of left intraventricular thrombosis and oral anticoagulant treatment options.

In the ventricle, thrombus formation reflects the presence of factors that represent Virchow’s triad, namely local myocardial injury, stasis of blood flow due to reduced wall motion and dilation of the heart chamber, and hypercoagulability (Figure 1). However, the contribution of endothelial dysfunction, inflammation, fibrosis, and stasis to thrombus formation in the LV can undergo variations related to the etiological substrate, and these differences may influence the response to the anticoagulant treatment [6]. Moreover, the anticoagulants may be part of dual or triple antithrombotic therapy and the antithrombotic effect could be potentiated by the summative effect of the drugs. This could be reflected in the speed and rates of thrombus resolution, but in the bleeding risk as well.

Most data related to LV thrombosis come from patients with acute myocardial infarction, especially those with anterior localization and akinetic apex that provide the perfect milieu for thrombosis. However, LV thrombus complicates both ischemic and non-ischemic cardiomyopathies, and there is little evidence of the latter. DOACs are currently used off-label for left ventricular thrombus owing to a paucity of evidence. For the same reason, there is a lack of guideline indication as well.

Evaluating the efficiency and safety of DOACs compared to VKA by including all patients with LV thrombi has the advantage of an analysis of a large number of cases but is hampered by the uneven distribution of the etiological substrate. Our work aims to fill this gap. Based on an exhaustive analysis of the available literature, we provide a structured and detailed update on the use of DOACs in particular clinical scenarios. As far as we know, this is the first paper that analyzes DOACs in this approach.

## 2. The Use of DOACs in Patients with LV Thrombus

### 2.1. Acute Myocardial Infarction

Early LV thrombosis is a common complication of acute myocardial infarction (MI), especially in patients with anterior or extensive ST-elevation myocardial infarction (STEMI) and a reduced LV ejection fraction. The cardiomyocytes’ ischemia and necrosis lead to wall motion abnormalities such as hypokinesia, akinesia, or dyskinesia that favor blood stasis within LV. Moreover, collagen exposure and subendothelial inflammation act as triggers for platelet aggregation and activation of coagulation [7]. 

In the first three months after an acute coronary event, the risk of LV thrombus occurrence and, consequently, cardioembolism, is the highest. The widespread use of percutaneous coronary intervention (PCI) in patients with STEMI has dramatically reduced the incidence of thromboembolic events related to LV thrombosis, from 22.3% before the PCI era to 5.5% in the PCI era [8]. The use of better ventricular anti-remodeling therapies, potent antiplatelet drugs, and dual antiplatelet therapy, as well as achieving a better time in therapeutic range (TTR) during warfarin anticoagulation, certainly substantially contributed to this decrease. 

Before DOACs were available, warfarin was the only oral anticoagulant used for the treatment of LV thrombosis post-acute MI. The minimum duration of the anticoagulant treatment is 3–6 months, and the imaging confirmation of thrombi resolution allows the discontinuation of anticoagulation [9,10]. However, this last aspect is still much debated, as case selection is very important and often very challenging. The therapeutic difficulty in LV thrombosis post-acute MI resides in the need for triple antithrombotic therapy, which entails an increased risk of bleeding. A preference for the use of DOACs over warfarin as part of the combined antithrombotic therapy is shown by the results of trials with DOACs in patients with atrial fibrillation and PCI. The addition of any of the four DOACs to a P2Y12 receptor inhibitor was associated with a similar or lower rate of hemorrhagic events and a similar rate of ischemic events compared to the triple therapy with VKA, a P2Y12 receptor inhibitor, and aspirin [11,12,13,14].

The successful use of DOACs in post-STEMI LV thrombosis has already been reported. The complete resolution of a 26 mm × 16 mm LV thrombus was obtained after 18 days of treatment with dabigatran added to dual antiplatelet therapy (DAPT) [15]. The resolution of a 40 mm × 14 mm LV thrombus was documented after 6 weeks of therapy with apixaban added to DAPT [16]. In a patient with two LV thrombi, rivaroxaban was added to DAPT for one month, then to single antiplatelet therapy (SAPT) until a six-month follow-up, when thrombus dissolution was confirmed [17]. Similar results come from a small series of patients, in whom the addition of rivaroxaban to DAPT led to complete resolution of thrombi at a 2–4-week follow-up [18]. In all these cases, DAPT was formed from aspirin and clopidogrel and neither ischemic nor thrombotic events were reported. In a patient with a 25 mm × 15 mm LV thrombus, rivaroxaban was added to DAPT represented by aspirin and a more powerful P2Y12 receptor inhibitor, namely ticagrelor. The complete thrombus resolution was confirmed at a three-month follow-up, in the absence of any hemorrhagic event [19]. The successful resolution of a small thrombus was achieved after one month of treatment with low-dose edoxaban and clopidogrel [20].

Since these first case reports, multiple evidence of efficiency and safety has been added. DOACs were used in both STEMI and non-ST-elevation myocardial infarction (NSTEMI) patients complicated with LV thrombosis, and in cases with available follow-up, the outcome was generally favorable. The thrombus resolution was confirmed in 82% of patients at a 1-year follow-up and in 86.1% of patients after a median follow-up period of 2.2 years [21]. The results of comparative studies between VKA and DOACs in patients with LV thrombus after anterior STEMI have recently been published. No significant difference in systemic thromboembolic events, major or minor bleeding, or the rate of thrombus resolution at three to six months of follow-up was found [22]. Of note, in 2014, apixaban, dabigatran, and rivaroxaban were introduced in the stroke prevention guideline as an alternative to VKA in patients with post-MI LV thrombus deemed unable to receive VKA due to non-hemorrhagic adverse events [23].

The most recent meta-analysis to date comparing DOACs with VKA included over 2000 patients with LV thrombus and showed that post-acute MI patients had a lower risk of stroke or systemic embolism, and bleeding if they used a DOAC and not VKA [24]. Fang et al. highlighted that DOACs might be superior to VKA for the treatment of post-acute MI LV thrombus. 

There are data suggesting that DOACs allow a quicker resolution of LV thrombus than VKA [21]. Still, it must be emphasized that there is no standardized protocol for monitoring patients with LV thrombus, so there is high heterogeneity between the reported results. Since the currently recommended duration of anticoagulant therapy is 3–6 months, it is expected that the first follow-up will be performed at the end of this time interval. One large study on acute MI patients reported that at the first follow-up, thrombus resolution was achieved in 70.7% of patients treated with a DOAC and 48.3% of patients treated with VKA [21]. 

The full thrombus resolution was documented in some patients after more than one year of treatment [25]. In other patients, the size of the thrombus remained unchanged, and it was necessary to change the VKA to a DOAC or vice versa. However, it was shown that treatment switching does not change either the embolic or hemorrhagic risk, or the thrombus resolution rate [26]. Although the vast majority of available data are concordant regarding the higher efficiency and safety of DOACs compared to VKA in patients with post-acute MI LV thrombus, there are case reports of thrombi that are difficult to resolve, do not reduce their size, or even form under anticoagulation. In a patient with STEMI treated by PCI, an LV thrombus formed under a triple antithrombotic regimen (aspirin, clopidogrel, and full-dose dabigatran) and was then complicated with a peripheral ischemic event on the 15th day of treatment [27]. This shows that there are still many unknown aspects that need to be studied.

Since nowadays much emphasis is placed on prevention, the role of DOACs has been studied in this setting as well. Low-dose rivaroxaban was added to standard DAPT in patients with anterior STEMI treated by primary PCI and it reduced the LV thrombi formation at a 30-day follow-up [28].

### 2.2. Myocarditis

Myocarditis is an inflammatory disease of the myocardium, usually resulting from common viral infections with cardiac tropism. Adenoviruses, enteroviruses, and herpesviruses are most commonly involved [29]. However, the etiological spectrum is much wider, and besides viruses, bacteria, fungi, parasites, and protozoa, a substantial number of non-infectious causes have been identified. Toxins, hypersensitivity reactions to various drugs, and immunological syndromes are among the myocarditis etiologies. 

Viral myocarditis evolves in three stages. First, the viruses enter cardiomyocytes and activate the innate immune response. Second, viral replication occurs and activates the acquired immune response. Finally, the evolution might mean a full recovery or the development of dilated cardiomyopathy [29]. While some viruses enter heart cells and cause myocyte necrosis and activation of the immune system, others affect the cardiac endothelial cells and trigger ischemia and systolic dysfunction by damaging the endothelium [29]. Active inflammation, which characterizes acute myocarditis, leads to ventricular dysfunction and a prothrombotic state. Due to the coexistence of abnormalities in LV parietal kinetic with excessive activation of coagulation, intracavitary thrombosis is common. Thromboses with varying degrees of severity have been reported, from LV mural thrombi with a low thromboembolic risk to biventricular thromboses with mobile or pedicled thrombi with a high thromboembolic risk [30,31,32] (Table 1). 

In animal models, viral myocarditis was associated with an increased myocardial tissue factor expression and activity [33]. Proinflammatory cytokines produced during viral infections were considered responsible for the increase in tissue factor expression in endothelial cells and monocytes [34,35]. Considering that there is cross-talk between the coagulation cascade and the inflammatory response, as long as the inflammation persists, there will be an activation of coagulation and an increased risk of thrombosis [35]. 

Eosinophils are granulocytes with roles in inflammation and immunological responses. After activation, eosinophil degranulation takes place, with the release of proteins involved in the production of free radicals, cell necrosis, and the induction of apoptosis [36]. Eosinophilic myocarditis has a three-stage evolution [37]. Firstly, eosinophils infiltrate cardiac tissue and release granular proteins that induce cardiomyocyte necrosis [38]. Secondly, mural thrombi form in areas with disrupted endothelial lining. Thirdly, fibrosis occurs, affecting the cardiac endothelium and valves. 

The thrombotic stage occurs when the mean duration of eosinophilia is 10 months [38]. The process has multiple contributors. Endothelial disruption exposes tissue factor, collagen, and von Willebrand factor, leading to coagulation activation on the surface of the denuded myocardium of the ventricular wall. This process is markedly enhanced by eosinophils that release tissue factor from their granules and stimulate the endothelium to express it [39,40]. Moreover, the proteins released by eosinophils bind to thrombomodulin and block its function [41]. Thrombomodulin can no longer bind circulating thrombin, leading to a hypercoagulable state and thrombosis. Direct activation of coagulation factor XII and platelets by proteins released from eosinophils and enhanced procoagulant activity of mononuclear cells were highlighted as well [42,43]. Along the damaged endocardium, mural thrombi are formed, usually involving both ventricles, the ventricular outflow tracts, and sub-valvular regions [38].

Current guidelines recommend initial treatment with unfractionated heparin (UFH) or low-molecular-weight heparins (LMWH) and bridging with VKA, with warfarin as the antithrombotic therapy of choice for intracardiac thrombi. Although the minimum duration of VKA treatment is three months [8], the length of anticoagulant treatment should be determined by the activity of the patient’s endomyocardial disease [38]. In a young man with myocarditis and concomitant left ventricular, right atrial, and pericardial thrombi, the one-month warfarin anticoagulant treatment led to full resolution of intracardiac thrombi and marked the regression of the intrapericardial thrombus [44]. Still, in a patient with eosinophilic myocarditis and intraventricular microthrombi, warfarin treatment resulted in a significant decrease in microthrombi but not complete resolution at a six-month follow-up [45]. Another patient had a fine rim of organized thrombus with a low risk for systemic embolization at a nine-month follow-up [46]. 

**Table 1:** LV thrombus in patients with myocarditis.

Author, Year	Sex, Age	Substrate	Antithrombotic Treatment	Thrombus Location and Size	Thrombus Outcome	Method of Confirming the Resolution of the Thrombus
McGee et al., 2018 [47]	M, 44 y	Bacterial myocarditis, normal LV size and systolic function	Enoxaparin, then Apixaban 5 mg bid	NR	Resolution at 3-week follow-up	CMR
Sossou et al., 2019 [31]	M, 33 y	History of acute viral perimyocarditis (4 months), HF, LVEF ~ 30–35%, PE, bilateral occlusion of the superficial femoral, popliteal, peroneal, anterior and posterior tibial arteries	UFH, then Rivaroxaban 15 mg bid for 21 days and 10 mg od thereafter	Multiple biventricular pedunculated mobile thrombi, 20–30 mm	Free of any complication at follow-up	NR
Tran et al., 2020 [48]	F, 62 y	Idiopathic eosinophilic myocarditis, mid-apical inferior and mid infero-lateral hypokinesia, HF, LVEF = 41%, small pericardial effusion	Apixaban 5 mg bid	Apical, mobile, 27 mm × 15 mm × 14 mm	Resolution at 3-month follow-up, thrombus absent at 12-month follow-up	TTE
Dimitroglou et al., 2021 [32]	F, 40 y	Eosinophilic myocarditis *Strongyloides stercoralis* infection, PE, DVT, HF, LVEF = 33%	Rivaroxaban *	Extensive mural thrombi	Resolution of thrombi at 3-month follow-up	TEE, CMR
Bodagh et al., 2022 [46]	M, 76 y	Eosinophilic myocarditis HF, dilated LV, LVEF = 33%, sub-endocardial apical fibrosis, hyper-eosinophilia secondary to respiratory infection	Rivaroxaban 20 mg od	Apical 28 mm × 14 mm	Resolution of majority of the thrombus at 9-month follow-up	TEE
Cottet et al., 2022 [49]	F, 42 y	Influenza A myocarditis, cardiogenic shock, LVEF = 25%, severe RV systolic dysfunction	Rivaroxaban 20 mg od	LV: apical, pedunculated, 22 mm × 15 mm, RV: apical	Resolution of thrombi at 8-day follow-up, thrombus absent at 6-month follow-up	TTE

M = male; F = female; LV = left ventricle; RV = right ventricle; PE = pulmonary embolism; DVT = deep vein thrombosis; VTE = venous thromboembolism; HF = heart failure; LVEF = left ventricle ejection fraction; TTE = transthoracic echocardiography; TEE = transesophageal echocardiography; * = rivaroxaban initiated for VTE.

### 2.3. Hypertrophic Cardiomyopathies

Hypertrophic cardiomyopathy (HCM) is a myocardial disease with a genetic substrate, characterized by a particular pattern of LV hypertrophy. 

Atrial fibrillation is a common complication of HCM. Due to this association, patients with concurrent HCM and AF have an increased risk of developing atrial thrombi [50,51]. Considering the high thromboembolic risk, life-long oral anticoagulation is recommended in these patients regardless of the CHA_2_DS_2_-VASc score and even when sinus rhythm is restored [52,53]. Moreover, DOACs are preferred over VKAs [52]. 

However, the presence of AF is not mandatory for the onset of LV thrombosis. In a short series of five cases of HCM and LV thrombus, only one patient had AF [54]. Of note, four of them had an apical aneurysm.

Occasionally, patients with HCM and AF can simultaneously develop thrombi in LA and LV, thereby having an extremely high thromboembolic risk. In a patient with AF and a history of gastrointestinal bleeding while on warfarin, discontinuation of anticoagulant therapy resulted in thrombosis in both the left atrial appendage and LV. The ventricular thrombus was attached to the chordal apparatus of the posterior mitral valve [50]. In this patient, cardioembolism manifested as multiple cerebral infarctions. After one month of apixaban treatment, the LV thrombus was no longer present and no ischemic or bleeding events were reported during this short follow-up period. 

Based on the distribution of hypertrophy, there are several phenotypes, such as symmetric, asymmetric, apical, and focal [55]. Of morphological types of HCM, the apical form carries an increased thrombotic risk due to apical outpouching. The marked increase in the apical parietal thickness is followed by subendocardial ischemia, wall motion abnormalities, dilatation, and subsequent aneurysm formation [56]. 

Hypokinetic/akinetic and aneurismal areas are prone to thrombi formation and are associated with a high risk of thromboembolic events. One study investigating intracardiac thrombosis in patients with heart failure reported that all five patients with HCM and LV aneurysm also had LV thrombosis [57]. In a cohort of almost 2000 patients with HCM, Rowin et al. found that 4.8% of patients (93) had an apical aneurysm [58]. Thrombi in the aneurysm were identified in 13 patients (14%) and another 5 patients suffered a nonfatal embolic event while in sinus rhythm and not receiving anticoagulant treatment. The aneurysms of these 18 patients were of all sizes, but large and medium-sized ones were more frequently encountered. Since thromboembolic events were twice more common in patients with apical aneurysms than in those without them, special attention should be paid to all patients with an apical aneurysm. Moreover, mid-ventricular obstruction can promote the development of an apical aneurysm, and thus close monitoring of these patients is necessary, as well [54]. 

LV thrombosis in HCM patients in sinus rhythm is a rare incidence. Still, a 40 mm apical thrombus was identified at the level of an LV apex aneurysm [59]. The presence of an apical aneurysm also explains the formation of thrombi in an LV with a normal ejection fraction [60]. A giant LV thrombus of 48 mm × 34 mm, covering a third of the LV cavity, was found in a patient with apical HCM, a restrictive pattern of diastolic dysfunction, and a preserved ejection fraction [60]. Fortunately, it entirely resolved after three months of warfarin treatment without ischemic or bleeding events. 

While patients with HCM and AF DOACs showed similar embolic and bleeding rates to VKA [61,62,63], little is known about their effectiveness and safety in patients with LV thrombus. Long-term oral anticoagulant treatment is indicated in patients presenting thrombi within the LV apical aneurysm [53]. Moreover, in all patients with apical aneurysms, an anticoagulant treatment can be considered. Unlike the cases with HCM and AF where DOACs are preferred over VKA, in patients with LV aneurysm with/without ventricular thrombus, the guideline does not express any preference over a class of anticoagulants [52]. In the largest study available to date, 18 patients with LV apical aneurysm and thrombi/thromboembolic events received anticoagulant treatment mainly with warfarin, and none experienced a thromboembolic event during follow-up [58]. Moreover, 25 patients with apical aneurysms received prophylactic anticoagulation and no ischemic event occurred during follow-up. In the entire cohort of 93 patients with apical aneurysms, 7 patients received DOAC, without any ischemic event. In a short series of five cases, LV thrombus resolution was achieved with apixaban (two cases), dabigatran (one case), and warfarin (two cases) [54] (Table 2). 

### 2.4. Tachycardia-Induced Cardiomyopathy 

The long-term action of elevated heart rate causes both diastolic and systolic LV dysfunction. The LV is dilated and has decreased contractility. Structural remodeling and myolysis are contributors to wall-thinning and the loss of contractile force. What is particular about tachycardia-induced cardiomyopathy is the reversible nature of dilated cardiomyopathy and the heart failure that it produces. In the case of a patient who presents both tachyarrhythmia and dilated cardiomyopathy with LV systolic dysfunction, the differential diagnosis between tachycardia-induced cardiomyopathy and tachycardias secondary to cardiomyopathy is extremely difficult and often possible only after heart rate control [65]. 

There are few case reports on LV thrombus in this setting [66,67,68]. In one patient, biventricular large and mobile thrombi were identified, requiring surgical treatment. Six months after the conversion of atrial flutter to sinus rhythm, the echocardiography was normal [66]. Another case is of a patient with congenital heart disease who presents atrial flutter, severe LV systolic dysfunction, and LV thrombus. Although he required intensive care and prolonged mechanical resuscitation, the LV thrombus dissolved under UFH and at the two-month follow-up the LV ejection fraction (LVEF) was normal [67]. Our search found only one case report of the use of DOAC in patients with tachycardia-induced cardiomyopathy. It is a patient with paroxysmal supraventricular tachycardia, severe LV systolic dysfunction, and an apical thrombus [69]. Thrombus size remained unchanged after six days of treatment with UFH and warfarin but dissolved completely after seven days of rivaroxaban. After treating the arrhythmia, the LVEF also improved, from 15% to 45%. 

### 2.5. Takotsubo Cardiomyopathy

Takotsubo cardiomyopathy is a stress-induced heart disease characterized by sudden and usually transient regional LV systolic dysfunction. The adrenaline surge leads to coronary spasm and microcirculation dysfunction, resulting in acute myocardial impairment. The echocardiographic feature is “apical ballooning”, the result of the abnormal motion of the apical and midventricular walls that appear akinetic or dyskinetic compared to the basal segments [70]. There is evidence that at the presentation, the plasma levels of catecholamine exceed those of patients with acute myocardial infarction [71]. Since the density of β-adrenoceptors is higher at the LV apex, the cardiomyocytes in this region are the most sensitive to excessive levels of catecholamines. At the apex, cardiomyocyte injury is the greatest, which explains the frequent location of thrombi at this site. A systematic review of 26 clinical trials found that in 94% of cases, the location of ventricular thrombosis was apical [72]. LV contractile function usually returns to normal after 4–8 weeks [73]. 

Furthermore, platelet and coagulation cascade activation and myocardial inflammation are present in the acute phase. Sometimes, persistent low-grade inflammation has been identified, and it is assumed that it may contribute to long-term cardiac dysfunction [74]. A significant reduction in endothelial function has also been identified, but its impact on short- and long-term outcomes is not yet clear [75]. 

LV thrombus is rare, occurring in only 1–2% of cases (Table 3). Patients with severe LV dysfunction, extended “apical ballooning”, and elevated troponin or inflammatory markers were most at risk [76,77]. When assessing ventricular thrombus and/or embolism in the acute phase of the disease, the percentage increased to 3.3% [78]. In a cohort of 95 patients with Takotsubo cardiomyopathy, LV thrombus was documented in 5 (5.3%) cases, all with an apical location [79]. LV thrombus may be identified at the initial presentation or may develop at any time later over the course of the disease [80,81]. In a series of 52 patients with Takotsubo cardiomyopathy, 4 of them had LV thrombi, identified at the time of diagnosis in 3 cases and 1 week later in the last case [81]. New evidence highlights that the thrombotic risk is highest in the first two days after the onset of Takotsubo cardiomyopathy [78]. LV thrombus represents 2–8% of complications during hospitalization [76].

When LV thrombus is present, patients with Takotsubo cardiomyopathy should receive anticoagulant treatment for at least three months or until LV function is restored [70]. Before DOACs become available, anticoagulant treatment consisted of intravenous UFH or subcutaneous LMWH during hospitalization and warfarin at discharge. Resolution of the LV thrombus was documented between one week [80,82] and three to four months of treatment [83,84]. An analysis of 36 patients found that under this type of anticoagulant treatment, complete resolution of the thrombus was achieved in all cases, in a time interval that varied between 9 and 90 days, with an average of 31 days [72]. 

In 2018, the non-vitamin K antagonist oral anticoagulants were introduced in the International Expert Consensus Document on Takotsubo Syndrome as a therapeutic option in patients with LV thrombus and/or embolization [76]. Although there is little experience in this direction, they have proven to be effective and safe [85,86]. No residual thrombosis was identified at the six-week follow-up, and no thromboembolic or hemorrhagic events were recorded during anticoagulation.

**Table 3:** LV thrombus in patients with Takotsubo cardiomyopathy.

Author, Year	Sex, Age	Substrate	Antithrombotic Treatment	Thrombus Location and Size	Thrombus Outcome	Method of Confirming the Resolution of the Thrombus	Comments
Kumar et al., 2021 [85]	F, 42 y	Severe global hypokinesis with apical akinesis, HF, LVEF = 17%, 5-FU treatment	Apixaban 2.5 mg bid, Aspirin 81 mg od	Apical, NR	Resolution at 6-week follow-up	TTE	Resolution of HF, LVEF = 70% Treatment stopped after 3 months
Blazak et al., 2022 [86]	F, 65 y	Circumferential akinesis of the mid to apical segments with hyperkinetic basal segments, fibromuscular dysplasia, type 2A spontaneous coronary artery dissection involving the first diagonal artery, HF, LVEF = 29%, RVEF = 49%	Rivaroxaban 15 mg od, Clopidogrel 75 mg od	Apical, 8 mm × 8 mm × 6 mm	Resolution at 6-week follow-up	Contrast-enhanced TTE	Resolution of LV dysfunction (LVEF = 56%) and normal RV size and function

F = female; LV = left ventricle; RV = right ventricle; HF = heart failure; LVEF = left ventricle ejection fraction; RVEF = right ventricle ejection fraction; TTE = transthoracic echocardiography; 5-FU = 5-fluorouracil; NR = not reported.

### 2.6. Left Ventricular Thrombus in Patients with COVID-19 

During the last three years of the COVID-19 pandemic, an important number of reports of thrombotic and thromboembolic events were collected, especially venous thromboembolism. COVID-19-associated coagulopathy is very complex and multifactorial. It partially overlaps but never perfectly matches with any of the following: sepsis-induced coagulopathy, disseminated intravascular coagulation, hemophagocytic syndrome, hemophagocytic lymphohistiocytosis, antiphospholipid syndrome, and thrombotic microangiopathy [87]. Acute coronary events, stroke, and acute lower-limb ischemia are the most common forms of arterial involvement. The risk of arterial thrombotic events is higher during the first week of infection, and then it sharply decreases [88]. However, recurrent thrombosis due to inflammatory flares after COVID-19 is possible [89]. 

To date, LV thrombosis in COVID-19 patients was associated with acute MI [90,91,92] and established cardiac disease [93,94,95], but also with the absence of any cardiac history both in young patients [96,97] and in elderly patients [98]. One study reported that among 368 patients admitted with COVID-19 and assessed by TTE during hospitalization, 4 had LV thrombus [99]. The presence of LV thrombus was always associated with regional wall motion abnormalities. In patients with concomitant acute MI and COVID-19, due to the intense states of hypercoagulability, LV thrombus may occur more frequently and much sooner than anticipated, and therefore these patients deserve special attention. 

Extreme thrombotic events have been reported in COVID-19 patients. A 17-year-old patient, otherwise healthy, experienced heart failure with reduced ejection fraction (HFrEF) and LV thrombus in the context of a severe multisystem inflammatory syndrome associated with COVID-19 [100]. Similarly, a 3-year-old child presented severe LV dysfunction and apical thrombus that required surgical removal due to high mobility and an increased risk of systemic embolism [101]. In another patient, an in-transit thrombus extending into the LV outflow tract and protruding through the aortic valve was identified [102]. A catastrophic clinical presentation was seen in a 57-year-old patient who developed heparin-induced thrombocytopenia associated with recurrent LV thrombosis, arterial thrombi in both lower limbs, and STEMI [103].

The high thrombotic risk determined by COVID-19 is reflected by the case of a 30-year-old woman with HFrEF who developed recurrent thrombosis in the LV and bilateral arterial embolism of lower limbs while on a therapeutic dose of apixaban, one month after infection with SARS-CoV-2 [104]. The treatment was successful, and the patient was discharged on warfarin, with a good outcome at the two-month follow-up. We also mention the case of a patient who developed a thrombus in an LV with normal wall motion [105]. 

There are few reports of successful use of DOACs in patients with LV thrombus and acute or recent SARS-CoV-2 infection (Table 4). Still, the available data suggest that DOACs can be a therapeutic option, especially since no hemorrhagic events have been reported.

### 2.7. Overview Studies and Meta-Analyses

These studies include a larger number of cases and allow for estimating the rate and speed of thrombi resolution, the prevalence of ischemic and hemorrhagic events during treatment with DOACs, and a comparative analysis with VKA.

A meta-summary of 36 case reports of DOAC use in patients with LV thrombosis found that thrombus resolution was achieved in 87.9% of cases, after a median duration of treatment of 30 days [107]. This thrombi resolution rate was similar to that of patients treated with VKA and with a TTR of over 50%. Only 41.7% of cases had acute MI as an underlying disease; therefore, this analysis provides an overview of the efficiency and safety of DOACs in various clinical scenarios. Rivaroxaban, dabigatran, and apixaban were used in 47.2%, 27.8%, and 25.0% of patients, respectively. No embolic events were recorded and only one non-fatal bleeding event occurred. 

An extensive literature search and analysis of the available data found that thrombus resolution was achieved in 75%, 86.2%, and 90% of patients receiving dabigatran, rivaroxaban, or apixaban, respectively [108]. Complete LV thrombus resolution was observed after a median duration of 28, 36, and 90 days for dabigatran, apixaban, and rivaroxaban, respectively. Adverse events were very rare: two ischemic and one hemorrhagic event. Of the three DOACs analyzed, apixaban treatment was not associated with either ischemic or hemorrhagic events.

In the last two years, small retrospective studies comparing DOACs with VKA published their results. Some showed similar rates of LV thrombus resolution, systemic thromboembolic events, and bleeding events with DOACs compared to VKA [109,110]. In a cohort of 84 patients with LV thrombus, 64 receiving VKA and 22 a DOAC, clinically significant bleeding events occurred only in patients receiving VKA [110]. In patients with heart failure and LV thrombus, rivaroxaban had a similar effect on thrombus resolution and bleeding risk as VKA, but a lower rate of major adverse cardiovascular events and systemic embolism [111]. One study reported that using a DOAC, the resolution of the LV thrombus is faster than with warfarin, with a median time of thrombus resolution of 32 days [112]. 

The results of three randomized clinical trials were recently published. Rivaroxaban 20 mg od was assessed in patients with newly diagnosed LV thrombus and compared to warfarin. Thrombus resolution was better with rivaroxaban than warfarin at all follow-ups, especially at a 1-month follow-up (71.79% vs. 47.5%) [113]. Rivaroxaban treatment performed better than warfarin to all endpoints. No strokes or systemic embolism occurred in the rivaroxaban group, while six patients had ischemic events in the warfarin group. Fewer bleeding events were seen in the rivaroxaban group than in the VKA group. In patients with LV thrombus identified in the first two weeks after an acute MI, apixaban was non-inferior to warfarin in thrombus resolution [114]. Moreover, in the apixaban group, no major bleeding events were recorded. In a pilot study, a reduction in thrombus size was assessed in patients treated with apixaban vs. warfarin [115]. Apixaban showed similar efficacy and safety to warfarin. 

The most robust data are provided by several meta-analyses, and all reached a similar conclusion. A meta-analysis of 6 studies, including 408 patients on DOAC and 1207 patients on VKA, compared the efficacy and safety of DOACs with that of VKA in patients with LV thrombus and highlighted that thrombus resolution, embolic events, and bleeding events were similar between the 2 groups [116]. Herald et al. highlighted that DOACs were as safe and effective as a warfarin treatment. Moreover, the incidence of intracranial hemorrhage, gastrointestinal bleeding, and other hemorrhagic events requiring hospitalization was significantly lower with DOACs than with VKA [117]. The most recent meta-analysis to date comparing DOACs with VKA included 12 observational studies and showed that DOACs and VKA had similar rates of thrombus resolution, stroke or systemic embolism, and bleeding events [24]. It also highlighted that in post-acute MI patients, the treatment with DOACs might be superior to VKA for the treatment of LV thrombus. The meta-analysis of Li et al. confirmed previous results, and in addition, it showed that concomitant antiplatelet medication did not influence the risk of stroke or systemic embolism, thrombus resolution, and bleeding events [26]. 

## 3. Discussion

Thrombus usually forms in large LV with systolic dysfunction, in aneurysms, or in areas with wall motion abnormalities such as hypokinesia or akinesia. Although thrombosis in an LV with a normal ejection fraction is rare, this particular situation should not be forgotten or ignored [7,60]. The apex is the region most commonly involved in both ischemic and non-ischemic cardiomyopathy patients. For decades, warfarin was used to prevent systemic embolism in patients with LV thrombus. Studies have highlighted that it is important not only to initiate the anticoagulant treatment, but also to obtain a stable therapeutic effect [118]. It was shown that 18% of patients develop an embolic event during anticoagulant treatment if TTR is below 50%. Even achieving a better TTR does not sufficiently eliminate the embolic risk, as 2.9% of patients develop embolic events at TTR higher than 50%.

The difficulties of VKA treatment lie in the multiple food and drug interactions, the unpredictable anticoagulant effect, the narrow therapeutic range, the need for frequent monitoring of the anticoagulant effect through blood tests, and the slow onset of the effect, which requires bridging therapy. DOACs are an enticing alternative to VKA because they do not have these shortcomings. Moreover, DOACs perform better than VKA in different clinical settings [119]. Currently, DOACs are preferred over VKA in AF patients eligible for a DOAC [1].

A dilated LV with parietal kinetic abnormalities is an environment characterized by low flow and low-shear stress, which is favorable for thrombosis. The same hemodynamic condition that predisposes to the formation of thrombi in the LV is also found in the left atrial appendage. Starting from this similarity, from the favorable outcomes in patients with AF [119], and from the evidence that DOACs lead to the dissolution of atrial thrombi [2,3,4], the premises for the use of DOACs in patients with LV thrombus were outlined. 

The mechanism of action could be an advantage of DOACs over VKA. Rivaroxaban prevents thrombosis but may favor the dissolution of established thrombi by direct inhibition of free and thrombus-bound factor Xa [120]. Rivaroxaban can reduce platelet activation [121] and induce a modification of the fibrin network [122]. A looser plasma fibrin network with thicker fibers and larger pores is more permeable to flow, and therefore more sensitive to fibrinolysis. Similarly, apixaban may enhance endogenous fibrinolysis [123]. Moreover, there is also evidence that dabigatran increases spontaneous thrombolytic activity by decreasing the expression of tissue factor pathway inhibitor (TAFI) and plasminogen activator inhibitor 1 (PAI-1) [124].

It is well-known that between inflammation and thrombosis exists a bidirectional relationship [125]. Inflammation induces the expression of tissue factor by monocytes under the action of interleukin-6 (IL-6), decreases the antithrombin levels, impairs the protein C system, and determines an insufficient inhibitory action of TFPI. Moreover, IL-6 enhances platelet production and activation. It was recently highlighted that LMWHs are capable of anti-inflammatory effects by reducing IL-6 levels [126]. Similarly, in patients with deep vein thrombosis, the treatment with dabigatran or edoxaban resulted in a 2.8 times reduction of the IL-6 expression levels in the peripheral lymphocytes [127]. The anti-inflammatory potential was identified in the case of rivaroxaban as well [128]. Lower levels of fibrinogen and inflammatory biomarkers were found in patients treated with rivaroxaban compared to the conventional approach consisting of LMWH and VKA. In an in vitro model, apixaban exhibited anti-inflammatory and antioxidant properties and prevented endothelial dysfunction [129]. In light of this recent evidence, the favorable anticoagulant effect of DOACs on LV thrombi could be the result of the amplification of their action by the associated anti-inflammatory effect. 

The data gathered so far show that DOACs are an alternative to VKA, having a rate of resolution of thrombi, and ischemic and hemorrhagic risks at least equal to that of VKA (Table 5). They have the advantage of a stable anticoagulant effect during treatment as well. Moreover, they perform similarly in a wide range of substrates. It was highlighted that the non-ischemic substrate and smaller thrombus area independently correlates with LV thrombus regression [130]. 

There were several reports of a lack of thrombus resolution and even thrombus formation in the LV during anticoagulant therapy, DOACs included [27,139,140]. Several hypotheses have been proposed to explain these events, including the variation of plasma drug concentration over 24 h for DOAC with bid administration, impaired absorption for dabigatran in the absence of adequate gastrointestinal acidity, and impaired liver metabolism for dabigatran, which is transformed from the prodrug into a drug under enzymatic action. Even the problem of different mechanisms of action of DOACs was raised. DOACs block the action of only one coagulation factor, Xa or IIa, which at least theoretically can lead to the upstream accumulation of coagulation factors, followed by the activation of coagulation in certain individuals. It could be enough for a thrombus to maintain its size or form [141]. However, in the absence of comparative studies, the superiority of one DOAC over another cannot be asserted.

Our study has several limitations arising from the studied literature. Firstly, only one DOAC has been studied in randomized clinical trials for this indication. Most data come from case reports and retrospective analysis which are subject to inherent biases. Secondly, DOACs were used in various dosing regimens and often in combination with one or two antiplatelet agents. This heterogeneity makes it very difficult to identify the most appropriate therapeutic strategy for the treatment of LV thrombosis. Thirdly, the assessment of thrombus resolution was not performed at standardized time intervals, and therefore a comparison between DOACs regarding the time required for thrombus resolution remains a desideratum for future studies. 

## 4. Conclusions

The anticoagulant treatment is of utmost importance in patients with left ventricle thrombus to prevent disabilities and death resulting from systemic embolism. Our thorough analysis of the available literature showed that DOACs can be an option for the treatment of LV thrombus regardless of the substrate. Considering the considerable amount of evidence accumulated so far, we expect a change in the guidelines soon. Our study found that apixaban was prescribed at 2.5 mg/5 mg bid, dabigatran at 110 mg/150 mg bid, edoxaban at 30 mg/60 mg od, and rivaroxaban at 15 mg/20 mg od, depending on creatinine clearance, age, and weight. In a few patients with concomitant pulmonary embolism, anticoagulants were administered according to the related guideline specifications. Available recommendations advocate a treatment period of at least 6 months, but the optimal duration is not well-known. Further prospective studies are needed to better guide the treatment of these patients. 

## Figures and Tables

**Table 2:** LV thrombus in patients with hypertrophic cardiomyopathy.

Author, Year	Sex, Age (Year)	Substrate	Antithrombotic Treatment	Thrombus Location and Size	Thrombus Outcome	Method of Confirming the Resolution of the Thrombus
Kaku et al., 2013 [64]	M, 59 y	Mid-ventricular obstructive hypertrophic cardiomyopathy and apical aneurysm, VT, ICD	Dabigatran 150 mg bid	15 mm × 17 mm	Thrombus resolution at 3-week follow-up, thrombus absent at 4-week follow-up	TTE
Kolekar et al., 2015 [51]	M, 61 y	Dilated phase of hypertrophic cardiomyopathy, AF, VT, HF, stroke, -CrCl = 71.31 mL/min	Dabigatran 110 mg bid	23 mm × 11.6 mm	Thrombus resolution at 1-month follow-up	TTE
Kaya et al., 2016 [50]	F, 60 y	Hypertrophic cardiomyopathy, AF, left atrium appendage thrombus, TIA, HF, LVEF 30%	Apixaban 5 mg bid	30 mm × 20 mm	Thrombus resolution at 1-month follow-up	TTE
Hamada, 2019 [54]	NR, 78 y	Hypertrophic cardiomyopathy, apical aneurysm	Apixaban	NR	Thrombus resolution	NR

M = male; F = female; NR = not reported; VT = ventricular tachycardia; ICD = implantable cardioverter-defibrillator; AF = atrial fibrillation; HF = heart failure; TIA = transient ischemic attack; LVEF = left ventricle ejection fraction; TTE = transthoracic echocardiography; TEE = transesophageal echocardiography; CrCl = creatinine clearance.

**Table 4:** Left ventricular thrombus in patients with COVID-19.

Author, Year	Sex, Age	Substrate	Antithrombotic Treatment	Thrombus Location and Size	Thrombus Outcome	Method of Confirming the Resolution of the Thrombus
Farouji et al., 2020 [93]	M, 60 y	HFrEF, LV hypertrophy	Enoxaparin 1 mg/kg bid, 7 days, then Apixaban 10 mg bid, 7 days, then 5 mg bid	LV thrombus of 30 mm × 30 mm	Reduction in size to 10 mm × 10 mm at 6-week follow-up	Recommendation of anticoagulation for 6 months, then TTE reevaluation
Jariwala et al., 2021 [102]	M, 67 y	STEMI, small LV apical aneurysm, LVEF = 33%, DM	DAPT and Enoxaparin 1 mg/kg, bid, 7 days, then Dabigatran 150 mg bid	Apical, 40 mm × 33 mm	Resolution at 30-day follow-up	TTE
	M, 45 y	AMI, LVEF = 40%, small LV apical aneurysm, de novo DM	DAPT and Enoxaparin 1 mg/kg bid, then Apixaban 2.5 mg bid	Apical, 30 mm × 18 mm	Resolution at 30-day follow-up	NR
Karikalan et al., 2022 [106]	F, 43 y	HF, LVEF = 25%, HTN, DM, stroke	Antiplatelets, Heparin during hospital stay, then DOAC	Mural thrombus, 18 mm	Reduction in size to 15 mm at 1-month follow-up	TTE
Zibaeenezhad et al., 2022 [105]	M, 66 y	HTN, normal LVEF, without regional wall motion abnormality	Enoxaparin, then Apixaban 5 mg bid	19 mm × 11 mm attached to anterolateral papillary muscles	Reduction in size at 10-day follow-up	TTE

M = male; F = female; LV = left ventricle; HFrEF = heart failure with reduced ejection fraction; STEMI = ST-elevation myocardial infarction; LVEF = left ventricle ejection fraction; DAPT = dual antiplatelet therapy; DM = diabetes mellitus; DOAC = direct oral anticoagulant; AMI = acute myocardial infarction; HF = heart failure; HTN = hypertension; TTE = transthoracic echocardiography; NR = not reported.

**Table 5:** Major studies assessing the efficacy and safety of DOACs in patients with LV thrombus.

Author, Year	Number of Patients on Anticoagulant Treatment					Main Outcomes DOACs vs. Warfarin
	A	D	E	R	W	
Cohort studies						
Ali, 2020 [131]	13	1	-	18	60	Rate of thrombus resolution (*p* = 0.85) Stroke (*p* = 0.33)
Cochran, 2020 [116]	Total of 14				59	Rate of thrombus resolution (*p* = 0.499) Stroke (*p* = 0.189) Bleeding (*p* = 1)
Daher, 2020 [109]	12	1	-	4	42	Thrombus resolution (*p* = 0.9) Stroke or systemic embolism (*p* = 0.8)
Guddeti, 2020 [132]	15	2	-	2	80	Thrombus resolution (*p* = 0.9) Stroke (*p* = 0.49) Bleeding (*p* = 0.96)
Iqbal, 2020 [110]	8	1	-	13	62	Thrombus resolution (*p* = 0.33) Stroke (*p* = 0.55) Systemic embolism (*p* = 0.55) Clinically significant bleeding (*p* = 0.13)
Robinson, 2020 [133]	141	9	-	46	300	Thrombus resolution (*p* = 0.64) Risk of stroke or systemic embolism was higher with DOACs vs. warfarin (*p* = 0.01)
Bass, 2021 [134]	79	29	-	77	769	New onset thromboembolic stroke (*p* = 0.13) Stroke or systemic embolism (*p* = 0.53) Bleeding (*p* = 0.40)
Iskaros, 2021 [135]	Total of 61				62	Thrombus resolution (*p* = 0.298) Shorter time to thrombus resolution with DOACs vs. warfarin (*p* = 0.003) Stroke or systemic embolism or bleeding (*p* = 0.213)
Jones, 2021 [21]	15	-	2	24	60	Greater and earlier LV thrombus resolution with DOAC vs. warfarin at 1 year (*p* = 0.0018) Major bleeding (*p* = 0.030) Systemic embolism (*p* = 0.388)
Willeford 2021 [136]	4	-	-	18	129	Thrombus resolution (*p* = 0.37) Stroke or systemic embolism (*p* = 0.37) Composite outcome (thrombus persistence, stroke, or systemic embolism) (*p* = 0.25) Bleeding (*p* = 1)
Xu, 2021 [137]	-	9	-	16	62	Thrombus resolution (*p* = 0.057) Stroke (*p* = 0.158) Systemic embolism (*p* = 0.906) Bleeding (*p* = 0.858)
Zhang, 2022 [138]	-	-	-	33	31	Thrombus resolution (*p* = 0.096) Quicker resolution with DOAC vs. warfarin (*p* = 0.049 at 6 months; *p* = 0.044 at 12 months; *p* = 0.045 at 18 months) Systemic embolism (*p* = 0.305) Bleeding (*p* = 0.444)
Randomized clinical trials						
Abdelnabi, 2021 [113]	-	-	-	39	40	Stroke (*p* = 0.08) Systemic embolism (*p* = 0.25) Bleeding (*p* = 0.11)
Haniff 2021 [115]	14	-	-	-	13	Reduction in thrombus size (*p* = 0.816) Similar safety outcomes
Alcalai, 2022 [114]	18	-	-	-	17	Thrombus resolution (*p* = 0.026 for non-inferiority)

A = apixaban; D = dabigatran; E = edoxaban; R = rivaroxaban; W = warfarin.

## Data Availability

Data sharing is not applicable to this article.
